# Circularly Polarized Reconfigurable MIMO Antenna for WLAN Applications

**DOI:** 10.3390/s25041257

**Published:** 2025-02-19

**Authors:** Tu Le-Tuan, Thai Dinh Nguyen, Nguyen Viet-Duc Tran, Hung Tran, Dat Nguyen-Tien

**Affiliations:** 1 Faculty of Electrical and Electronic Engineering, PHENIKAA University, Yen Nghia, Ha Dong, Hanoi 12116, Vietnam; thai.nguyendinh@phenikaa-uni.edu.vn (T.D.N.); nguyen.tranvietduc@phenikaa-uni.edu.vn (N.V.-D.T.); dat.nguyentien@phenikaa-uni.edu.vn (D.N.-T.); 2 Department of Convergence Engineering for Intelligent Drone, Sejong University, Seoul 13391, Republic of Korea; huyhung@sejong.ac.kr

**Keywords:** circularly polarized, polarization reconfigurable, MIMO, metasurface

## Abstract

This paper presents a simple design of a two-element antenna with circularly polarized (CP) reconfigurability for multiple-input multiple-output wireless local-area network (WLAN) applications. A MIMO element consists of a reconfigurable feeding network, a CP source, and a 2 × 2 unit-cell metasurface (MS). By controlling the ON/OFF state of PIN diodes, the proposed MIMO system can operate in either right-hand CP (RHCP) or left-hand CP (LHCP) for all ports, or either RHCP or LHCP for each port. For all operating modes, the proposed antenna exhibits good performance with a matching performance of less than –10 dB, an axial ratio of lower than 3 dB, as well as an inter-port isolation of better than 24 dB at 2.45 GHz. Additionally, the MIMO diversity performance is also satisfied by the proposed antenna. Compared to related works, the proposed antenna has advantages of high gain and compact size, as well as a simple switching mechanism with a small number of PIN diodes.

## 1. Introduction

In the scenario of modern wireless communications, it has been demonstrated that the system performance would be highly improved by the combination of a multiple-input multiple-output (MIMO) system and polarization reconfigurable antennas [1]. This type of configuration would effectively avoid fading loss thanks to the deployment of circularly polarized (CP) reconfigurable antennas. Further enhancements to the system benefit from MIMO technology, these being high data-rate transmission and high reliability.

Various types of CP reconfigurable antennas have been reported in the open literature [2,3,4,5,6,7]. These designs produce good characteristics in terms of polarization reconfigurability while offering stable radiation performance. In addition, their benefits are either a broad operating band [2,3] or compact dimensions [4,5]. In contrast, there are critical trade-offs regarding gain and bandwidth while switching between different modes. For MIMO systems, several designs in [8,9,10,11,12,13] have been presented. The proposals in [8,9,10,11,12] offer excellent isolation performance while providing stable reconfigurability across their operating BW. Meanwhile, the antenna in [13] can provide right-hand CP (RHCP) and left-hand CP (LHCP) radiations for each port within a compact and low-complexity design. In general, such antennas only work with fixed polarization states [8,9,10,11,12,13]. Meanwhile, the polarization reconfigurable antennas in [2,3,4,5,6,7] only work with single elements, which have not been developed for the MIMO system.

Several polarization reconfigurable MIMO antennas using monopole/slotted structures have been published in [14,15,16,17]. Such antennas are generally designed with multiple PIN diodes integrated into feeding lines to obtain polarization reconfigurability. However, low gain radiation is the most critical drawback of monopole or slotted structures due to their omnidirectional patterns. For higher gain, an MIMO antenna with a microstrip patch structure is presented in [18]. This antenna can provide multiple polarizations including RHCP, LHCP, and vertically and horizontally linear polarization (V-LP, H-LP). In general, the current polarization reconfigurable MIMO antennas have low gain radiation, complicated switching circuits and a necessity for multiple PIN diodes.

In this paper, an antenna system with the properties of a high gain, compact, and low complexity switching mechanism is proposed for MIMO systems. Instead of using a slot or monopole structure, a MS-based radiator is employed to achieve high gain and compact dimensions as well. Then, the polarization diversity is achieved by using a reconfigurable feeding network, which is located beneath the ground plane. The single MIMO element occupies only two PIN diodes attached to the feeding network, which would prevent the unwanted effects from degrading the overall performance. The MIMO configuration consists of two closely spaced elements, which can operate either in similar polarizations (RHCP or LHCP) or different polarizations (one in RHCP and the other in LHCP).

## 2. Design of MIMO Element

### 2.1. Passive Antenna

**Figure 1:**
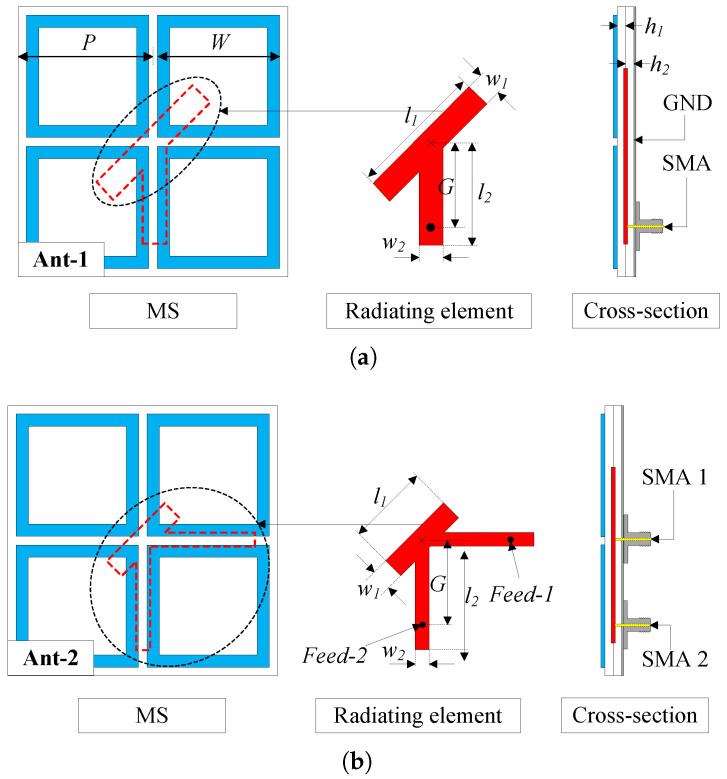
Geometry of the passive antennas. (**a**) 1-port antenna, and (**b**) 2-port antenna.

The geometrical configuration of the passive antenna in terms of top- and side-view is shown in Figure 1. The first antenna (Ant-1) is designed with a single port, while the second antenna (Ant-2) with dual-sense CP operation is a development from Ant-1. Both antennas are printed on two Taconic RF-35 substrates with a dielectric constant of 3.5 and a loss tangent of 0.0019. The metasurface (MS) layer consists of 4-unit cells arranged in 2 × 2 configuration, acting as a primary radiating aperture of the antenna. Meanwhile, a patch acts as a CP source, which has asymmetrical geometry to produce two orthogonal modes with equal magnitude and 90° phase difference. The details optimization process of these designs can be found in [19], which is not presented for brevity. The antenna is optimized using the full-wave High-Frequency Structure Simulator (HFSS) and the optimal dimensions of Ant-1 and Ant-2 are given in Table 1.

**Figure 2:**
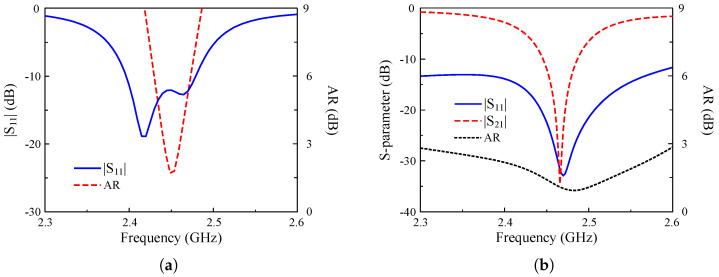
Simulated performance of (**a**) Ant-1 and (**b**) Ant-2.

The simulated performance of Ant-1 and Ant-2 in terms of reflection coefficient ($|S_{11}|$), axial ratio (AR), and transmission coefficient ($|S_{21}|$) is shown in Figure 2. The simulated data indicate that both antennas have good performance around 2.45 GHz with $|S_{11}|$ and an AR lower than –10 dB and 3 dB, respectively. For a two-port antenna (Ant-2), mutual coupling between the ports is very important to avoid degrading the system’s performance. Ant-2 observes high isolation between Feed-1 and Feed-2 of about 35 dB.

### 2.2. Polarization Reconfigurable Antenna

**Figure 3:**
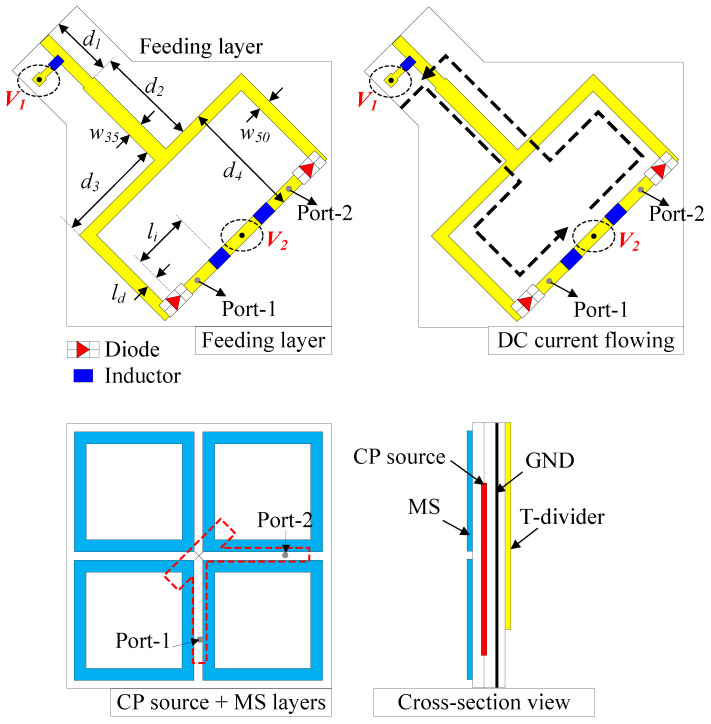
Geometry of the polarization reconfigurable antenna.

It is noted that the proposed polarization reconfigurable MIMO antenna in this paper is a further development work from [19]. Firstly, a polarization reconfigurable element is considered. According to the performance of Ant-2, high isolation between two ports is obtained. Thus, a polarization reconfigurable antenna is developed from this design. Here, the polarization switching mechanism is based on a reconfigurable feeding network. Figure 3 shows the geometrical configuration of the proposed polarization reconfigurable antenna. Here, Feed-1 and Feed-2 of Ant-2 are excited through a T-junction power divider. RHCP radiation will be achieved with Feed-1 excitation, while LHCP realization is obtained with Feed-2 excitation. The T-junction power divider is printed on a 0.8-mm-thick FR4 substrate. The optimized dimensions of the CP reconfigurable antenna are as follows: *P* = 22, *W* = 20.3, *w* = 1.9, $l_{1}$ = 12, $w_{1}$ = 3, *G* = 12, $w_{2}$ = 1.2, $l_{2}$ = 18, $w_{35}$ = 2.4, $w_{50}$ = 1.9, $d_{1}$ = 9.7, $d_{2}$ = 15.9, $d_{3}$ = 16.8, $d_{4}$ = 18.9, $l_{d}$ = 1.1, $l_{i}$ = 9.8 (unit: mm).

The polarization switching mechanism is based on two diodes $D_{1}$ and $D_{2}$, which are the type of MADP-042305-130600 manufactured by MACOM^TM^ in Lowell, MA, USA. The ON state of the diode is modeled with a resistor of 1.32 Ω, while the OFF state is equivalent to a 0.15 pF capacitor [20]. The diodes’ positions and their arrangements are as illustrated in Figure 3. As the PIN diodes act as a resistor in the ON state, the loss will be introduced, and it affects the radiation efficiency of the antenna. It should be noted that to minimize the impact of diodes on the radiation efficiency, low-loss PIN diodes should be a solution. Furthermore, locating PIN diodes as proposed in the paper is the best solution to avoid its negative effect on the radiation characteristic. Two biasing wires are connected to the divider at $V_{1}$ and $V_{2}$, which have different voltages to control the diodes independently. Three 220-nH inductors (MLJ1005WR22JT000) are utilized to block the RF current flowing from the divider to the biasing wire. According to the arrangement of the diodes, it can be clearly seen that when $V_{1}$ is smaller than $V_{2}$, $D_{1}$ will be activated and $D_{2}$ will be deactivated. At this time, the CP source is excited with Feed-1 and then, the RHCP wave will be radiated. On the other hand, $D_{2}$ will be turned ON and $D_{1}$ will be turned OFF with a $V_{1}$ greater than $V_{2}$, leading to the LHCP radiation due to the Feed-2 excitation of the CP source. The DC current flowing for different biasing voltage condition ($V_{1}$ > $V_{2}$ and $V_{1}$ < $V_{2}$) is indicated as dot-black curves in Figure 3.

**Figure 4:**
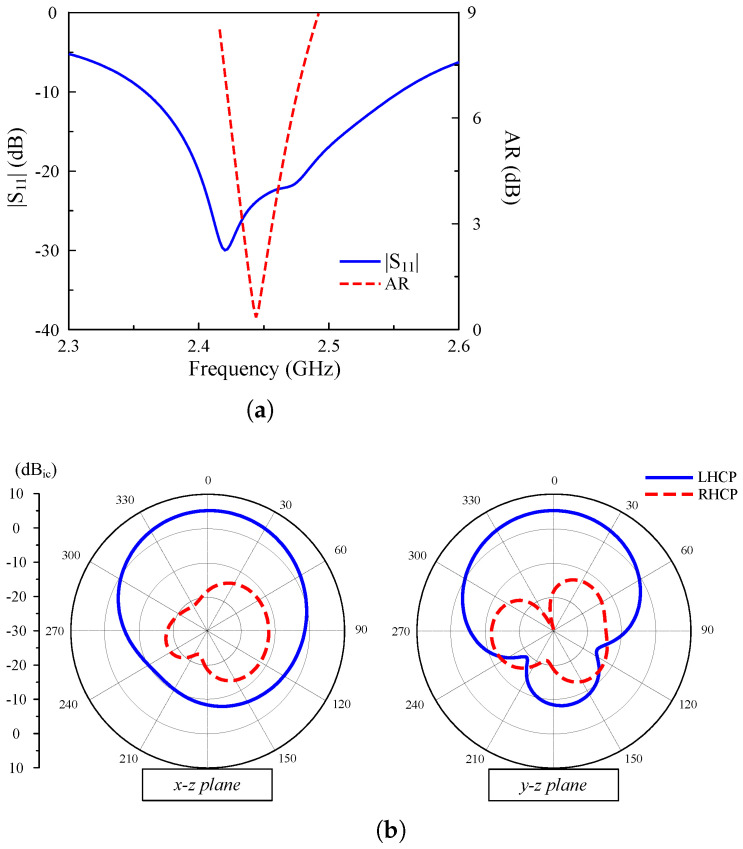
The simulated performance of the polarization reconfigurable antenna. (**a**) $|S_{11}|$ and AR, (**b**) gain radiation patterns at 2.45 GHz.

**Figure 5:**
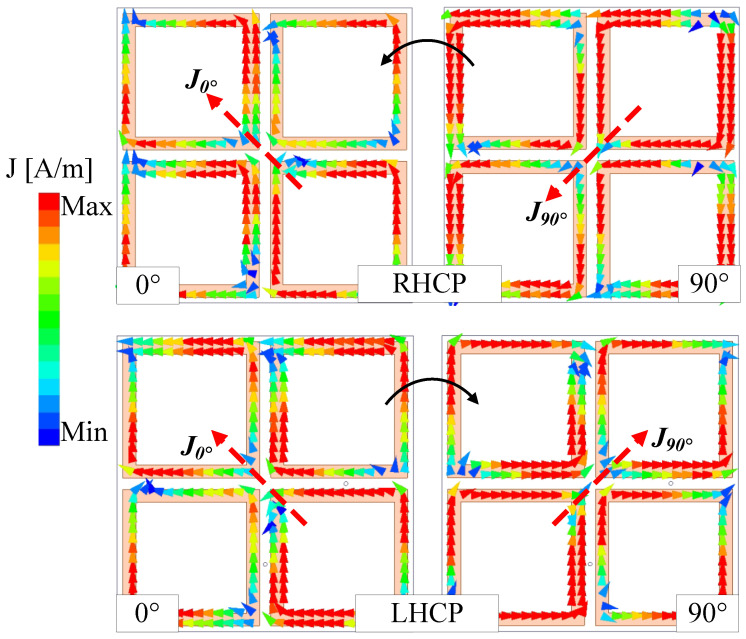
The simulated vector current on the MS layer at 2.45 GHz.

The performance of the reconfigurable antenna is shown in Figure 4. Due to the symmetrical configuration, the results for Feed-1 and Feed-2 excitations are identical. Thus, only the simulated results with Feed-1 excitation are presented. It can be seen clearly that the antenna exhibits good operation characteristics around 2.45 GHz. The reflection coefficient is about 25 dB, while the AR is close to 0. In terms of gain radiation patterns, they are quite symmetric around the broadside direction. The broadside gain value is about 5.0 dBi. Due to the small size, the back radiation of the antenna is quite high at about –8 dB. The CP realization of the reconfigurable antenna is verified by the current distributions. Figure 5 shows the simulated vector current on the primary radiating MS layer at 2.45 GHz for different excitations. As observed, when the phase changes from 0° to 90°, the vector current of the RHCP state (Feed-1 excitation) rotates in the counter-clockwise direction. In contrast, the rotated direction is clockwise for the LHCP state (Feed-2 excitation).

## 3. Design of Polarization Reconfigurable MIMO Antenna

### 3.1. MIMO Configuration

**Figure 6:**
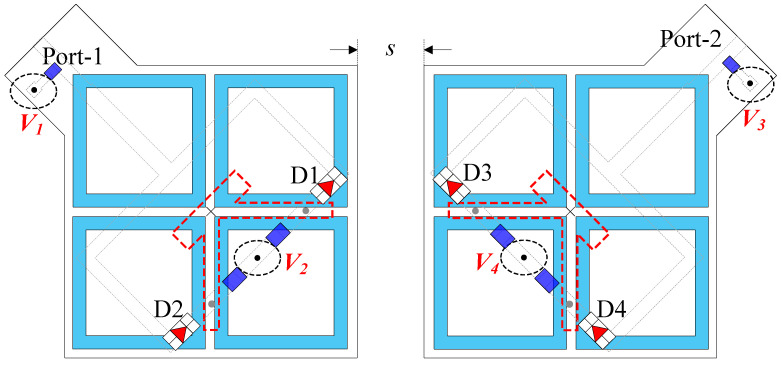
Geometry of the proposed CP reconfigurable MIMO antenna.

Figure 6 shows the geometry of the proposed polarization reconfigurable MIMO antenna. This antenna is developed from the CP reconfigurable antenna presented in Section 2.2. Two elements are positioned at a distance of *s* = 5 mm. Here, four PIN diodes designated as $D_{1}$, $D_{2}$, $D_{3}$, and $D_{4}$ are employed, and their arrangements are as demonstrated in Figure 6. These diodes are biased by voltages $V_{1}$, $V_{2}$, $V_{3}$, and $V_{4}$. The polarization switching mechanism of each MIMO element is like the discussion in the previous Section.

### 3.2. Operating Performance

Each MIMO element can switch its polarization between the RHCP and LHCP radiation. In the MIMO configuration, both ports can operate with either similar polarizations or different polarizations. The diode states and corresponding polarization states of the proposed antenna are summarized in Table 2.

**Figure 7:**
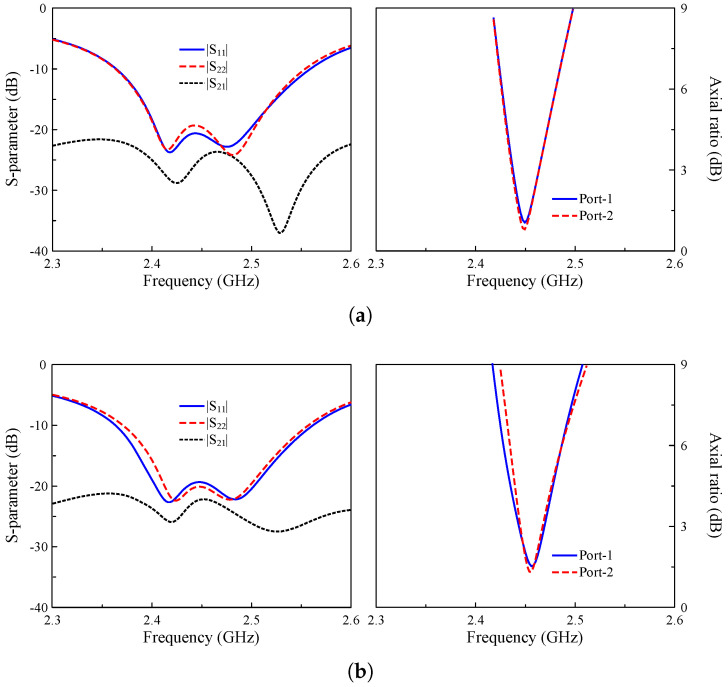
Simulated performance for (**a**) Case-1 and (**b**) Case-2.

The simulated performance in terms of reflection and transmission coefficients ($|S_{11}|$ and $|S_{21}|$), and the AR for both ports, is shown in Figure 7. It should be noted that due to the symmetrical geometry, the results are quite similar; thus, only the simulated data for Case-1 and Case-2 are presented. Obviously, all operating cases are well resonated at 2.45 GHz. Wide impedance matching bandwidth is achieved for all cases. Meanwhile, the overlapped 3-dB AR bandwidth of all cases is about 20 MHz. With respect to isolation, it is always better than 22 dB across the operating spectrum.

**Figure 8:**
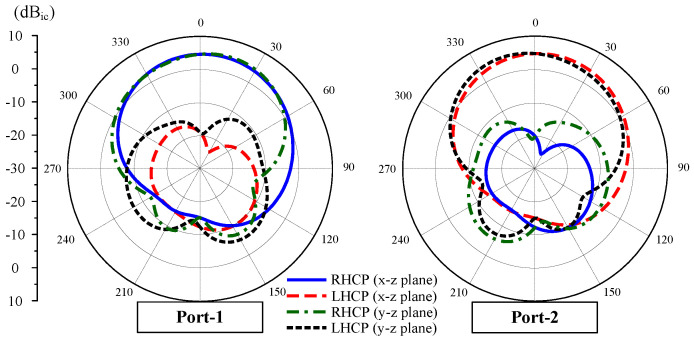
Simulated gain radiation patterns at 2.45 GHz for Case-1.

In terms of gain radiation patterns, Figure 8 shows the results in two principal planes of x-z and y-z for Case-1. In general, good radiation characteristics are observed for both ports. The antenna has a broadside radiation pattern. The dominant radiation for Port-1 is RHCP, while that for Port-2 is LHCP.

## 4. MIMO Diversity Performance

This section validates the diversity performance of the proposed antenna. The evaluation is based on several major parameters, which are Envelope Correlation Coefficient (ECC), Diversity Gain (DG), and Mean Effective Gain (MEG).

**Figure 9:**
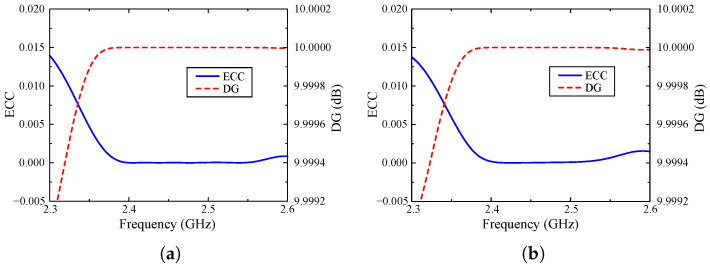
Calculated ECC and DG of different cases. (**a**) Case-1 and (**b**) Case-2.

The first parameter to be considered when evaluating the diversity performance of a MIMO antenna is ECC, which is defined as the correlation between each MIMO element when considering them as transmitting and receiving units. Equation (1) describes the method to obtain the values of ECC by using the antenna’s S-parameters. In addition, the calculation of DG figures indicates how much the signal-to-noise ratio (SNR) improves when the multi-element antenna is deployed, which is provided by Equation (2). As observed, ECC and DG clearly have an inverse relationship. Consequently, when the MIMO elements exhibit low correlation, the obtained DG will be high. The values of ECC must meet the requirement of lower than 0.5, while the peak threshold for DG is 10 dB. As illustrated in Figure 9, where the ECC and DG of both Case-1 and Case-2 are outlined, the proposed antenna offers outstanding diversity performance within its operating band. Accordingly, the value of ECC is consistently lower than 0.15, which is far more lower than the required threshold of 0.5. Furthermore, the changes in DG curve are extremely small around the predefined peak value of 10 dB.

(1) $$ECC=\frac{{\left|S_{ii}^{*}*S_{ij}+S_{ji}^{*}*S_{jj}\right|}^{2}}{\left(1-{\left|S_{ii}\right|}^{2}-{\left|S_{ji}\right|}^{2}\right)\left(1-{\left|S_{jj}\right|}^{2}-{\left|S_{ij}\right|}^{2}\right)}$$

(2) $$DG=10\sqrt{1-{\left(ECC\right)}^{2}}$$

**Figure 10:**
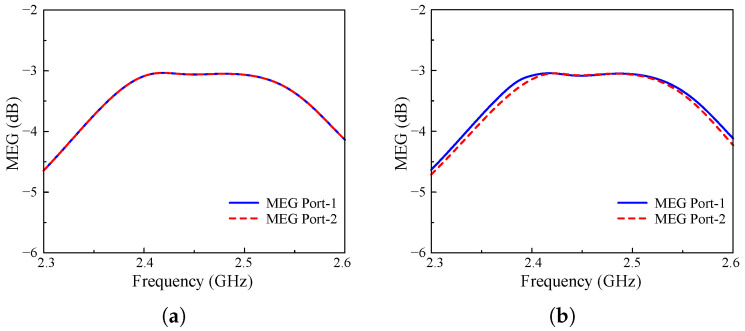
Calculated MEG of different cases. (**a**) Case-1 and (**b**) Case-2.

Next, MEG values are carried out via S-parameters, as shown in Equation (3). It is important to note that when taking into account the spatial distribution of incoming signals and the multipath environment, MEG measures the average power received by an antenna in comparison to an isotropic antenna. The optimal threshold of MEG is determined as lower than −3 dB while the curves for each element should be uniformly illustrated. Therefore, the difference between each MIMO element in terms of MEG should be less than 3 dB, as listed in Equation (4). Figure 10 depicts the simulated MEG values, which are calculated in both cases for each MIMO port. As described, the two ports of the proposed antenna perform identical MEG, with the same curves in Case-1 and a slight difference for Case-2. Hence, it could be concluded that the proposed antenna would yield a MEG magnitude difference that would be very close to 0.

(3) $$MEG_{i}=0.5\left[1-\sum_{j=1}^{N}{\left|S_{ij}\right|}^{2}\right]$$

(4) $$\left|MEG_{i}-MEG_{j}\right|\leq 3\;\mathrm{dB}$$

## 5. Measurement Results

**Figure 11:**
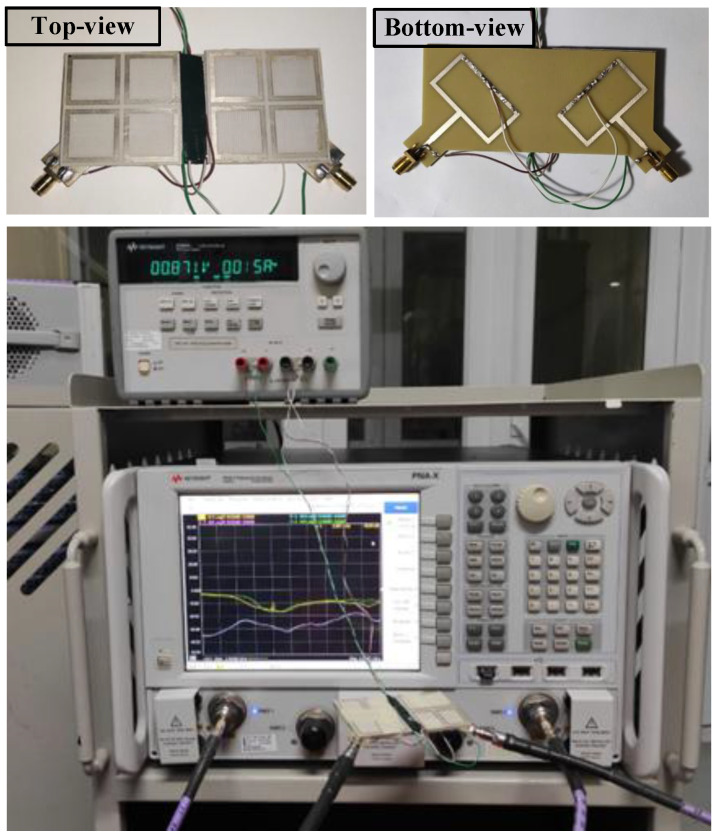
Photographs of the fabricated MIMO antenna.

To validate the simulated results, an antenna prototype is fabricated and measured. The photographs of the prototype and measurement setup are shown in Figure 11. The operation characteristics in terms of reflection coefficient and transmission coefficient are validated by Keysight Vector Network Analyzer N5242A manufactured by Keysight Technologies, Inc., Santa Rosa, CA, USA with a testing frequency up to 26.5 GHz. Meanwhile, the antenna’s axial ratio as well as gain radiation patterns are tested using an Anechoic Chamber. The measurement setup includes the use of a Keysight DC Power supply E3634A to correctly bias the diodes, with a typical biasing voltage of 0.87 V and a current of 10 mA. Overall, the measured data are well-matched with the simulated ones.

**Figure 12:**
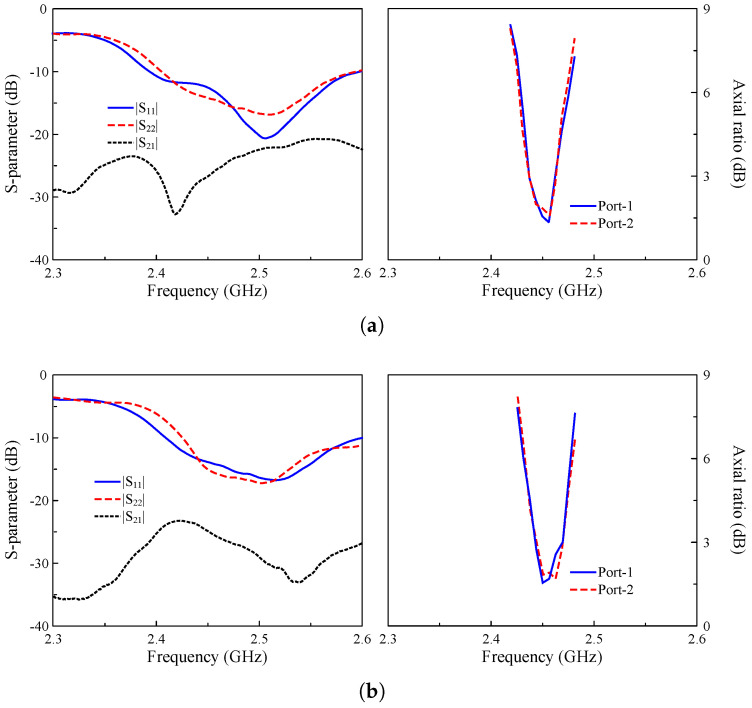
Measured S-parameter and AR of the proposed antenna. (**a**) Case-1 and (**a**) Case-2.

**Figure 13:**
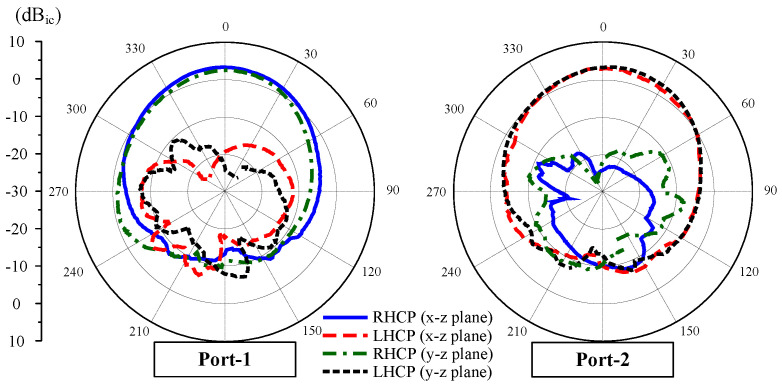
Measured gain radiation patterns at 2.45 GHz for Case-1.

Figure 12 shows the simulated and measured scattering parameters and AR when the MIMO system operates in Case-1 and Case-2. The measured impedance bandwidth covers the 2.45 GHz band. Meanwhile, the isolation around 2.45 GHz is always better than 24 dB for all cases. Regarding the CP radiation, the MIMO antenna exhibits good CP radiation around 2.45 GHz with AR values of less than 3 dB from 2.44 to 2.46 GHz. The gain radiation patterns of Port-1 in Case-1 and Case-2 are plotted in Figure 13. As seen, the broadside radiation pattern is achieved for all cases with the broadside gain of about 4.3 dBi. The antenna radiates RHCP when Port-1 is excited and LHCP with Port-2 excitation.

## 6. Comparison

To demonstrate the advantages of the proposed work, Table 3 summarizes the performance comparison between the proposed antenna and the other related works. Overall, it is obvious that the proposed antenna has a compact size, high gain radiation, and simple polarization switching mechanism with a small number of PIN diodes. The slot antennas in [14,15,16] feature low gain radiation due to their omnidirectional radiation patterns. Additionally, the antenna in [15] is able to operate with LP radiation. Although high gain can be achieved in [17,18], large size and/or large number of PIN diodes are their critical drawbacks.

## 7. Conclusions

The simple design of two-element MIMO antenna with CP reconfigurability for WLAN applications is proposed and investigated in this paper. The polarization switching mechanism is based on four PIN diodes, which are integrated into the T-junction power dividers. By turning ON or OFF these PIN diodes, the MIMO system can operate with the same polarization for all ports, or either RHCP or LHCP for each port. The measured data demonstrate that all operating cases have good performance at 2.45 GHz. Furthermore, the MIMO diversity performance is also investigated to ensure the suitability of the proposed antenna in the MIMO system. Accordingly, the proposed CP reconfigurable antenna can be a potential candidate for the WLAN systems.

## Figures and Tables

**Table 1:** Optimal dimensions of Ant-1 and Ant-2 (unit: mm).

Parameters	Ant-1	Ant-2	Parameters	Ant-1	Ant-2
*P*	22	22	*G*	12.4	12
*W*	20	20.3	$l_{2}$	13.4	18
$l_{1}$	13.2	12	$w_{2}$	4	1.2
$w_{1}$	3.8	3	$h_{1}$	1.52	1.52
$h_{2}$	1.52	1.52			

**Table 2:** Summary of pin diode states and polarization states of each MIMO element.

Operating Case	Diode States				Polarization State	
	$D_{1}$	$D_{2}$	$D_{3}$	$D_{4}$	**Port-1**	**Port-2**
Case-1	ON	OFF	ON	OFF	RH-CP	LH-CP
Case-2	ON	OFF	OFF	ON	RH-CP	RH-CP
Case-3	OFF	ON	ON	OFF	LH-CP	LH-CP
Case-4	OFF	ON	OFF	ON	LH-CP	RH-CP

**Table 3:** Performance comparison among polarization reconfigurable MIMO antennas.

Ref.	Antenna Size (λ)	Antenna Type	No. of Elements	No. of Diodes	Polarization	Max. Gain (dB)
[14]	0.22 × 0.22 × 0.01	Slot	2	2	RHCP, LHCP, LP	1.1
[15]	Not Given	Slot	2	4	V-LP, H-LP	2.2
[16]	1.14 × 0.79 × 0.03	Slot	3	10	RHCP, LHCP	2.6
[17]	1.25 × 1.36 × 0.03	Monopole	4	4	RHCP, LHCP, LP	4.5
[18]	1.22 × 1.22 × 0.05	Patch	2	16	RHCP, LHCP, LP	5.8
Prop.	0.76 × 0.76 × 0.03	MS	2	4	RHCP, LHCP	4.3

## Data Availability

Data are contained within the article.
